# Supplementary material for: Impact of acute decompensation on the prognosis of patients with hepatocellular carcinoma
Source: PLoS One. 2022 Jan 27;17(1):e0261619. doi: 10.1371/journal.pone.0261619 (PMC8794202; doi:10.1371/journal.pone.0261619)
Supplement: Attachment — Submitted filename: Rebuttal letter.docx [file pone.0261619.s002.docx]

**PONE-D-21-23680R1
Impact of acute decompensation on the prognosis of patients with hepatocellular carcinoma
PLOS ONE**

**To the Reviewers**

**Thank you for your comments and critical review.**

Reviewer #1: The article greatly improved by the corrections made by the authors. I have still some minor comments:

- How do the authors explain that cirrhosis is not a risk factor for AD or prognosis when compared to non-cirrhotics?

**Reply: Thank you for your comments. In our study, cirrhosis was associated with AD development (marked-up copy; page 9, lines 17-18; page 16, line 11). However, cirrhosis is not a risk factor for long-term mortality. Therefore, we added some sentences for explaining this result in the Discussion section (marked-up copy; page 15, lines 6-11).**

- Part of the discussion is on why Albi-score and CLIF-C are associated with prognosis, but if I understand it correctly these were predictors of prognosis in the original study and not in the rebuttal. If this is the case, the authors should focus on discussion of the new predictors if they think this is needed ( ACLF, bilirubin level, HCC progression or MELD score)

**Reply: We agree with your suggestion. According to your suggestion, we deleted some sentences in the Discussion section (marked-up copy; page 16, lines 20-25; page 17, line 1).**

- They authors suggest to perform surgical resection in patients who have HCC with preserved liver function, even at the intermediate-advanced–stage, because of the low AD incidence rate after treatment. The fact that AD is low in the resection group can also be reflected by the relative good status of patients in this group (younger age, no portal hypertension, few comorbidities). If this is the case, it is possible that the low AD occurence in the surgical group is attributed to the patient group and not to the intervention (selection bias). Did the authors compare baseline characteristics of HCC paitents in the different groups to evaluate this? If these groups are indeed different, the authors should consider to remove this conclusion.

**Reply: Thank you for your comments. According to your comments, we compared baseline characteristics between the surgical and non-surgical groups. In fact, patients who received surgery had lower Child-Pugh scores than those who did not, and had no prior history of AD. Therefore, we modified some sentences in the Abstract section (marked-up copy; page 3, lines 14-15, 22-24)** **and the Discussion section (marked-up copy; page 14, lines 22-23; page 17, lines 10-13).**

- Please check the manuscript for spelling errors, the word cirrhosis is spelled incorrectly in some placed (for example methods section abstract)

**Reply: We apologize for some errors. We checked and corrected some errors in the Abstract section (marked-up copy; page 3, lines 9, 18), the Patients and Methods section (marked-up copy; page 7, line 23), the Results section (marked-up copy; page 9, line 16; page 12, lines 17-18, 25), and the Discussion section (marked-up copy; page 14, line 21).**

Reviewer #2: I would like to thank the authors for their efforts to respond to all our comments. The changes applied to the manuscript improved its quality subtantially.

Authors should go through the manuscript once more to remove some remaining typos.

**Reply: We would also like to thank you for taking the time to review our manuscript and apologize for some remaining typos. We checked and corrected the remaining typos in the Abstract section (marked-up copy; page 3, lines 9, 18), the Patients and Methods section (marked-up copy; page 7, line 23), the Results section (marked-up copy; page 9, line 16; page 12, lines 17-18, 25), and the Discussion section (marked-up copy; page 14, line 21).**
